# A Computational Model of the Escape Response Latency in the Giant Fiber System of *Drosophila melanogaster*

**DOI:** 10.1523/ENEURO.0423-18.2019

**Published:** 2019-04-15

**Authors:** Hrvoje Augustin, Asaph Zylbertal, Linda Partridge

**Affiliations:** 1 Institute of Healthy Ageing, and GEE, University College London, London WC1E 6BT, United Kingdom; 2 Max Planck Institute for Biology of Ageing, Cologne D-50931, Germany; 3 Department of Neuroscience, Physiology and Pharmacology, University College London, London WC1E 6BT, United Kingdom

**Keywords:** aging, computational model, *Drosophila*, escape response, gap junctions, ion channels

## Abstract

The giant fiber system (GFS) is a multi-component neuronal pathway mediating rapid escape response in the adult fruit-fly *Drosophila melanogaster*, usually in the face of a threatening visual stimulus. Two branches of the circuit promote the response by stimulating an escape jump followed by flight initiation. A recent work demonstrated an age-associated decline in the speed of signal propagation through the circuit, measured as the stimulus-to-muscle depolarization response latency. The decline is likely due to the diminishing number of inter-neuronal gap junctions in the GFS of ageing flies. In this work, we presented a realistic conductance-based, computational model of the GFS that recapitulates the experimental results and identifies some of the critical anatomical and physiological components governing the circuit’s response latency. According to our model, anatomical properties of the GFS neurons have a stronger impact on the transmission than neuronal membrane conductance densities. The model provides testable predictions for the effect of experimental interventions on the circuit’s performance in young and ageing flies.

## Significance Statement

Finding potential targets for preventing functional decline of neuronal circuits is important from both biological and clinical perspective. In the nervous system of *Drosophila melanogaster*, the escape response system mediates quick propagation of signals from the brain to the muscles, instructing flight initiation following a threatening visual stimulus. It was previously shown that this circuit shows a marked decline in the speed of signal propagation with age, likely due to loss of synaptic gap junctions. Here, we generated a computational model of the system and uncovered novel anatomic and physiologic parameters that govern the circuit’s function in young and old animals. These predictions can be tested experimentally and have significance for other fast circuits in flies and other species.

## Introduction

**Figure 1.**
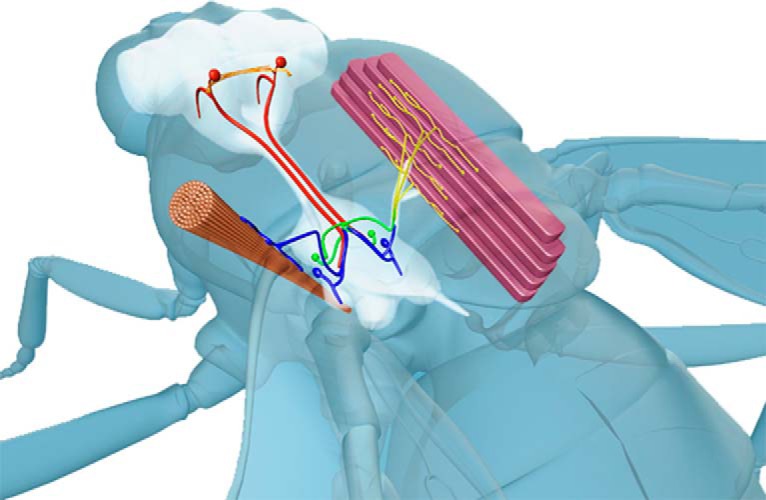
Diagram of the GFS anatomy. Two GF interneurons originating in the brain (red) descend to the thoracic ganglia where they connect, via a mixed (electrical and chemical) synapse, to the TTMn (blue) innervating the cylindrical TTM. In the second branch of the circuit, the GFs form a mixed synapse with the PSI (green), which, in turn, chemically synapses onto the DLMns (yellow) innervating the DLMs. Red circles in the brain denote approximate positions of the GF cell bodies.

Escape responses are evolutionarily ancient mechanisms used by many species as their main defense against predator attacks. Intense selection pressure has led to dedicated reﬂex circuits that continuously monitor the environment for danger and trigger escape behaviors when presented with a speciﬁc set of threatening stimuli. These circuits must be able to respond within a minimal time frame to prevent capture and maximize chances of survival (Herberholz et al., 2004; Walker et al., 2005). Escape circuits are therefore characterized by extremely fast reaction times, with response latencies as short as a few milliseconds (Card and Dickinson, 2008; Dill, 1974). In dipteran insects, escape responses are mediated by the giant fiber system (GFS). Prompted by a visual (and, possibly, mechano-sensory) stimulus, the adult fruit-fly *Drosophila melanogaster* executes a stereotyped sequence of events that results in an escape jump followed by flight initiation (Trimarchi and Schneiderman, 1995; Allen et al., 2006; Fayyazuddin et al., 2006). The GFS consists of two descending, non-myelinated giant fiber (GF) interneurons that originate in the brain, and downstream neurons that innervate and activate flight muscles (dorsal longitudinal muscles, DLMs) and jump muscles (tergotrochanteral muscles, TTMs; King and Wyman, 1980; Sun and Wyman, 1997; Allen et al., 2006; Fig. 1). A single a single action potential (AP) in a GF axon is sufficient to initiate patterned activity in jump and flight muscles (Koto et al., 1981). Functionally, electrical synapses are a dominant type of synapse in the *Drosophila* GFS (Phelan et al., 1996; Trimarchi and Murphey, 1997), with chemical (cholinergic) synapses playing a minor role (Allen and Murphey, 2007). Gap junctions are the physical substrate of electrical synapses that provide physical continuity between the cytoplasms of closely apposed pre- and post-synaptic neurons (Bennett, 1997). Compared to chemical synapses, transmission across electrical synapses is extraordinarily fast, with the possibility of the current flowing in either direction across the gap junction (Purves et al., 2001). Electrical synapses are therefore frequently found in places where fast transmission is critical, such as in escape response and motion-processing circuits (Cook and Becker, 1995). In the *Drosophila* GFS, the shaking-B gene (*shakB*, *inx8*) instructs the formation of heterotypic, unidirectional (rectifying) electrical synapses (Phelan et al., 1998; Stebbings et al., 2002; Wu et al., 2011).

Loss of gap junctions in the nervous system occurs normally as a consequence of aging. For example, astrocytic gap junctional plaques are drastically reduced in the brains of aging mice (Cotrina et al., 2001), likely affecting inter-astrocytic and neuron-glia metabolic coupling (Cai et al., 2017). The structural proteins comprising the gap junctional channels are called connexins or pannexins in vertebrates (Hormuzdi et al., 2004) and innexins in invertebrate species (Hasegawa and Turnbull, 2014). In various knock-out mutants, widespread disruption of the neuronal gap junctional coupling leads to reduced synchronicity of neural networks (Deans et al., 2001), impaired oscillatory patterns in the brain (Buhl et al., 2003), neuronal hyperexcitability (Sutor et al., 2000), increased neuronal apoptosis (Nakase et al., 2003), and reduced neuroprotection after ischemic injury (Siushansian et al., 2001).

Recently, Augustin et al. (2017) showed that the response latency through the GFS (i.e., the time between the stimulation of the GFs in the brain, and flight or jump muscle depolarization) increases with age, demonstrating an age-related decline in the functionality of the escape circuit. These experimental results suggest that the prolonged signal propagation is likely due to the age-associated decline in the conductance via gap junctions. This hypothesis is based on the findings that the old flies exhibited severely reduced ShakB plaque size (indicating reduced gap junctional volume and consequent attenuation of the junctional conductance), with other potentially contributing factors to this decline such as neuromuscular function and GF diameter being unaffected by age (Augustin et al., 2017). In this study, we generated a realistic computational biophysical model of the GFS based on these findings and on previously reported properties of the circuit’s components. By exploring potential determinants of response latency, including membrane properties, neuronal geometry and gap junction conductance, we created a model that not only recapitulates the previously reported experimental results, but also elucidates the relative importance of different physiologic and anatomic parameters in regulating the speed of signal propagation through this escape response circuit.

## Materials and Methods

### Code accessibility

The code described in the paper is freely available online at http://modeldb.yale.edu/245415. The code is available as Extended Data 1.

10.1523/ENEURO.0423-18.2019.ed1 Extended Data 1 Code for data acquisition, analysis, and generation of all figures. Download Extended Data 1, ZIP file.

### Model architecture

**Figure 2.**
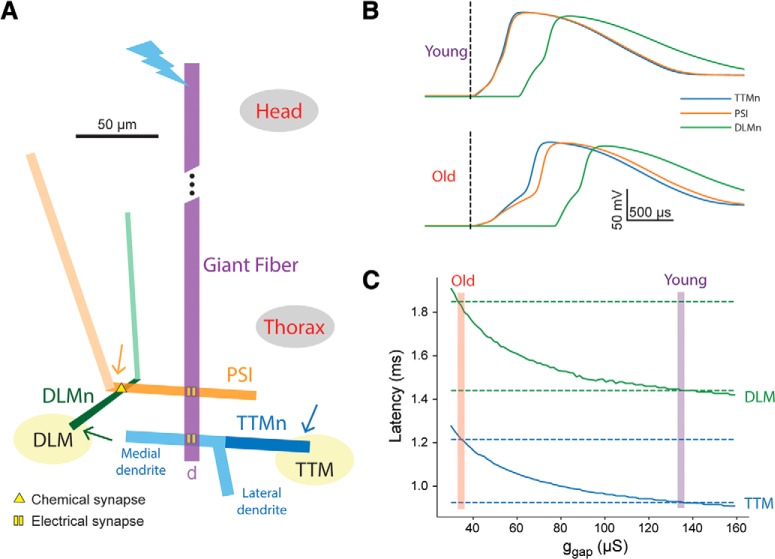
GFS model architecture and response latency measurements. ***A***, Model architecture and geometry, showing the cylindrical sections that make up the four cell types in the model (to scale), along with the location of electrical and chemical synapses. Active sections (axons) are shown in dark colors. Bolt denotes the proximal end of the GF that is stimulated in the simulation, and arrows denote the distal ends of the axons, from which the voltage recordings shown in ***B*** were taken. The response latency in the DLM pathway is slightly delayed compared to the latency in the (shorter) TTM branch. ***B***, Membrane potential recorded in the model TTMn (blue), DLMn (green), and PSI (orange), for “young fly” g_gap_ value (135 μS, top) and “old fly” g_gap_ value (34.5 μS, bottom). ***C***, Latency from stimulus onset to muscle response as predicted by the model for TTM (blue) and DLM (green), as a function of g_gap_. The latency values recorded experimentally are indicated by dashed lines, and the g_gap_ values where they coincide with the values predicted by the model are shown by magenta and red bars (for young and old flies, respectively).

To implement the model, we used the NEURON simulation environment with Python (Hines and Carnevale, 1997; Hines et al., 2009) ran on a Dell PC laptop using Ubuntu operating system. The model of the *Drosophila* GFS (of either sex) is comprised of four cells: The GF neuron, the TTM motoneuron (TTMn), a peripherally synapsing interneuron (PSI), and a DLM motoneuron (DLMn; Fig. 2*A*). Each neuron contains one to three unbranched cylindrical sections (functional subunits) with dimensions based on anatomic data (see model parameters below).

Each section is divided into 51 iso-potential segments, that form the basic computational unit of the model and connected via fixed specific axial resistance. The membrane potential in each segment is calculated as a function of time based on the cable equation and any fixed or time-varying membranal conductances it contains. The GF is modeled as a single active section that forms unidirectional electrical synapses onto the active section (axon) of the PSI and the medial passive section (dendrite) of the TTMn. The TTMn contains two dendrites (medial and lateral; Godenschwege et al., 2002a) and an active axon. The PSI contains a dendrite and an axon, which forms a chemical synapse onto the active section (axon) of the DLMn. The DLMn contains a tapering axon and a dendrite (King and Wyman, 1980; Egger et al., 1997).

### Model conductances

All model sections contain a passive leak conductance. Active sections (axons) were largely modeled according to an existing model of a *Drosophila* motoneuron (Günay et al., 2015) and based on Hodgkin–Huxley type channel kinetics (Hodgkin and Huxley, 1952). They contain persistent and transient voltage-gated sodium channels, as well as voltage-gated potassium channels, with kinetics based on Günay et al. (2015). Each conductance type is distributed with equal density in all active sections. The PSI-DLMn chemical synapse is modeled as a double-exponential process.

### Simulation

To test the TTM and DLM response latency in the model, we stimulated the proximal end of the GF with a current step duration of 0.03 ms (similar to that used by Augustin et al., 2017) and amplitude of 120 nA to approximate the input to the GF during high-amplitude head stimulation and measured the latency to the AP peak in the TTMn and DLMn. To compare this latency with the latency values measured experimentally, we added 0.35 ms to this value to account for the neuromuscular junction delay. This value was estimated from the experimentally measured “neuromuscular latency” of ∼0.65 ms. The neuromuscular latency is the time period between thoracic stimulation that directly stimulates the motoneurons, and TTM or DLM (muscle) depolarization (Augustin et al., 2017). This duration is the sum of two periods: (1) the time that passes from thoracic stimulus onset to AP peak in the TTMn or DLMn, and (2) the time from the AP peak to muscle depolarization (NMJ delay). To estimate the first part, the model TTMn was stimulated directly (simulating thoracic stimulation), resulting in ∼0.3 ms from stimulus onset to AP peak. The estimated NMJ delay is therefore the remaining 0.35 ms, achieving a total of 0.65 ms. This delay contributes a fixed bias to the latency values, and therefore plays no role in assessing the relative importance of model parameters.

### Model parameters

The model parameters were chosen according to known values from the literature, where available (Table 1). Some of these values were manually adjusted to make sure all the model cells are spiking, and the response latencies match recorded values. Dimensions in the simplified anatomy were chosen to capture the general proportions of the cells and the ratio between active and passive membrane area.

**Table 1.** Anatomic and physiologic parameters used in the article

Anatomical parameters	
GF diameter	8 μm (Augustin et al., 2017)
GF length	400 μm (Phelan et al., 1996; Smith et al., 1996)
Distance of contact with TTMn from proximal end	400 μm (distal end of the GF)
Distance of contact with PSI from proximal end	360 μm (King and Wyman, 1980; Phelan et al., 1996)
TTMn diameter	6 μm (King and Wyman, 1980)
TTMn axon length	50 μm (Godenschwege et al., 2002b)
TTMn medial dendrite length	60 μm (Godenschwege et al., 2002b)
TTMn lateral dendrite length	30 μm (Godenschwege et al., 2002b)
Distance of input from GF from medial dendrite proximal end	12 μm (Godenschwege et al., 2002a,b)
PSI diameter	4.5 μm (King and Wyman, 1980)
PSI axon length	90 μm (Phelan et al., 1996; Egger et al., 1997)
PSI dendrite length	170 μm (estimated)
Distance of input from GF from PSI axon proximal end	45 μm (Egger et al., 1997; Blagburn et al., 1999)
Distance of contact with DLMn from PSI axon proximal end	76.5 μm (Egger et al., 1997)
DLMn dendrite and axon proximal diameter	2 μm (King and Wyman, 1980)
DLMn axon distal diameter	4 μm (King and Wyman, 1980)
DLMn axon length	50 μm (Sun and Wyman, 1997)
DLMn dendrite length	100 μm (Sun and Wyman, 1997)
Distance of input from PSI from axon proximal end	12.5 μm (Egger et al., 1997)
Physiological parameters	
Leak conductance	0.03 mS/cm^2^ (estimated)
Specific membrane capacitance	1 μF/cm^2^ (estimated)
Specific axial resistance	35.4 Ω/cm (estimated)
Maximal transient voltage-gated sodium conductance (ḡ_Nat_)	300 mS/cm^2^ (Günay et al., 2015)
Maximal persistent voltage-gated sodium conductance (ḡ_Nap_)	0.11 mS/cm^2^ (Günay et al., 2015)
Maximal voltage-gated potassium conductance (ḡ_K_)	10 mS/cm^2^ (estimated)
Gap junctions conductance (g_gap,_ young fly)	135 μS (estimated)
Gap junctions conductance (g_gap_, old fly)	34.5 μS (estimated)
Chemical synapse rise τ	0.1 ms (standard value)
Chemical synapse decay τ	1 ms (standard value)
Chemical synapse reversal potential	0 (standard value)
Chemical synapse delay	0.15 ms (estimated)
Chemical synapse peak conductance	80 μS (estimated)
Neuromuscular junction delay	0.35 ms (see Materials and Methods)
Leak reversal potential	–85 mV (Günay et al., 2015)
Sodium reversal potential	65 mV (Günay et al., 2015)
Potassium reversal potential	–74 mV (Günay et al., 2015)

## Results

### A conductance-based model of the *Drosophila* GFS reproduces aging-related latency increase

To examine how the electrical coupling in the fly’s GFS contributes to the transmission latency, we stimulated the model circuit with a 120-nA pulse at the proximal end of the GF (Fig. 2*A*) and recorded the voltage at the distal ends of the TTMn, PSI, and DLMn neurons. Setting the gap junction conductance (g_gap_) of all model electrical synapses to 135 μS resulted in the voltage recordings shown in Figure 2*B*, top. The latency from stimulus onset to AP peak, summed up with a fixed neuromuscular junction latency (0.35 ms), matches the values recorded experimentally in the TTM and DLM of young (5–7 d old) flies (0.93 and 1.44 ms, respectively; Augustin et al., 2017). This value of g_gap_ will therefore be used to model the response latency in young flies. When using this value, the latency in the PSI and TTMn cells is similar, owing to their equivalent position in the circuit (one electrical synapse away from the GF).

Decreasing g_gap_ to 34.5 μS results in longer membrane charging time to firing threshold due to weaker current across the gap junction, and thus to increased latency for both TTM and DLM, up to the values recorded in old (45–50 d old) flies (1.22 and 1.85 ms; Fig. 2*B*, bottom). This value of g_gap_ will therefore be used to model the response latency in old flies. When using this value, the latency in the PSI becomes larger compared to the TTMn, since at lower inward currents the differences in morphology between these cells play a bigger role (namely, the long dendrite of the PSI that increases the load on the input current). Scanning a range of conductance values reveals the expected monotonic decrease of the response latency as g_gap_ increases (Fig. 2*C*). The model therefore reproduces the time latencies from stimulus to jump and flight muscle depolarization and shows that a 4-fold reduction in gap junction conductance by itself could account for the transition from the latencies measured in young flies to the latencies measured in old flies.

### Co-dependency of the response latency on g_gap_ and other physiologic and anatomic parameters

**Figure 3.**
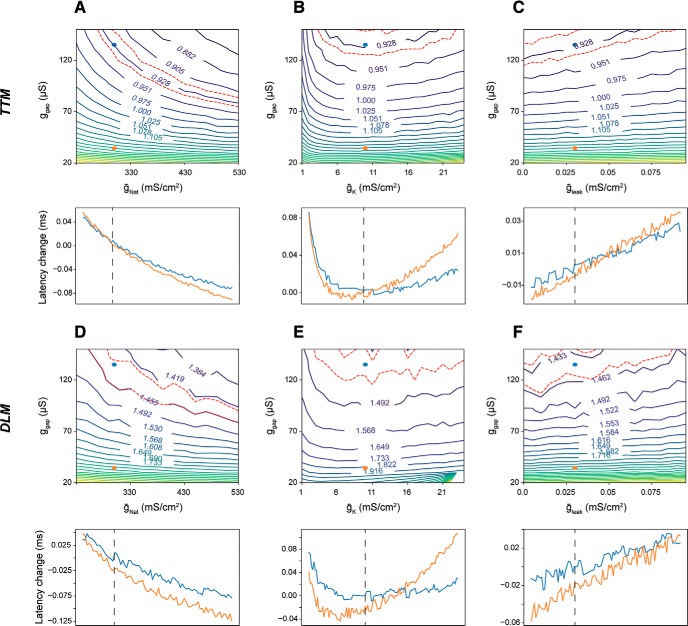
Co-dependency of the response latency on g_gap_. ***A–C***, top, The latency landscape in the TTM, shown using iso-latency lines (labeled with response latency values in milliseconds) as a function of the global gap junction conductance (g_gap_) and maximal transient voltage-gated sodium conductance (ḡ_Nat_, ***A***), maximal voltage-gated potassium conductance (ḡ_K_, ***B***), and leak conductance (ḡ_leak_, ***C***). Blue and orange dots represent the values for young and old flies, respectively. The region in the landscape representing young fly latency is marked by red dashed lines. Bottom, Cross sections in the latency landscape, showing the change in latency (relative to experimentally measured values) as a function of the three conductance types, for young flies (blue) and old flies (orange). ***D***–***F***, same as ***A–C***, for the DLM.

Next, we tested how the response latency predicted by the model changes as a function of both g_gap_ and membranal conductance densities. We performed two-dimensional grid scans by varying g_gap_ (spanning the values used for young and old flies), along with either the maximal transient voltage-gated sodium conductance for all axons (ḡ_Nat_; Fig. 3*A*,*D*), the maximal voltage-gated potassium conductance for all axons (ḡ_K_; Fig. 3*B*,*E*), or the leak conductance in all sections (g_leak_; Fig. 3*C*,*F*). The latency values are presented as contour maps, where regions in the parameter space with a similar latency as young flies (± 1%) are denoted by red dashed lines. For example, Figure 3*A*, top, shows that similar TTM response latency as that of young flies (0.928 ms) is achieved when a reduction in g_gap_ (moving toward the bottom) is compensated by an increase in ḡ_Nat_ (moving toward the right). The plots below the contour maps show the change in latency for young (blue) and old (orange) flies as a function of the conductance in question, thus they represent horizontal cross sections through the contour maps. While the directionalities of the effects on response latency were expected from basic biophysical principles, this approach enabled us to assess the relative efficacies of these changes. As expected, increased transient voltage-gated sodium conductance reduced the response latencies (Fig. 3*A*,*D*) due to lowered AP threshold, and to a lesser degree by faster AP propagation. The reverse effect was observed when increasing the potassium and leak conductances (Fig. 3*B*,*C*,*E*,*F*), since these changes shift the membrane potential away from firing threshold and shunt inward current, leading to an increase in the time needed to reach threshold during a pre-synaptic spike. An extreme reduction in potassium conductance also prolonged the latency (Fig. 3*B*,*E*, left side of the plot) by elevating the resting membrane potential and thus causing sodium conductance inactivation. Within the tested ranges of conductance values, no change in a single parameter reverted the latency of an old fly (orange dot) to that of a young fly.

**Figure 4.**
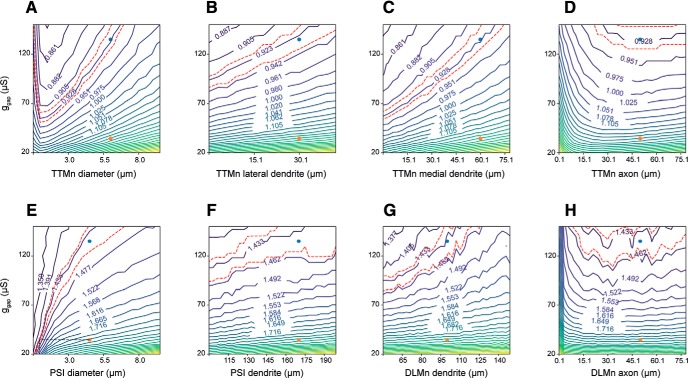
Impact of anatomic model parameters on response latency. ***A–D***, TTM Latency as a function of g_gap_ and anatomic parameters in the TTM branch of the model: the diameter of TTMn sections (***A***), and the length of the TTMn lateral dendrite (***B***), medial dendrite (***C***), and axon (***D***). ***E–H***, DLM latency as a function of g_gap_ and anatomic parameters in the DLM branch of the model: PSI section diameter (***E***), PSI dendrite length (***F***), DLMn dendrite length (***G***), and DLMn axon length (***H***).

We next tested how the response latency varied when changing g_gap_ along with anatomic parameters. For the TTMn branch of the circuit, we tested the following parameters: TTMn diameter (Fig. 4*A*), the length of lateral and medial TTMn dendrites (Fig. 4*B*,*C*), and the TTMn axon length (Fig. 4*D*). For the DLMn branch, we tested the PSI diameter (Fig. 4*E*), PSI dendrite length (Fig. 4*F*), DLMn dendrite length (Fig. 4*G*), and the DLMn axon length (Fig. 4*H*). Since increases in diameter and length of neuronal sections decrease the cells’ input resistance, these changes in general decreased the effect of a given input current on the membrane potential and thus prolonged the latency to response. Among the tested parameters, a ∼5-fold decrease in the PSI diameter reverted the response latency of an old fly to that of a young fly (Fig. 4*E*, black dashed line). Contrary to the general trend, where a reduction of section dimensions reduces the latency, a reduction in axonal length beyond a critical value prolonged the response latency due to the decrease in active membrane surface area (and thus, in total active conductance, Fig. 4*D*,*H*). Overall, changes in the diameter of neuronal section affected the membrane capacitance throughout the length of the section, making their influence on the latency stronger compared to changes in section length. The results from Figures 3, 4 show that, according to the model, changes in membrane conductance densities (within realistic limits) are far less efficient compared to anatomic changes in affecting the response latencies through the two branches of the GF circuit.

### Co-dependency of the response latency on other parameter combinations

**Figure 5.**
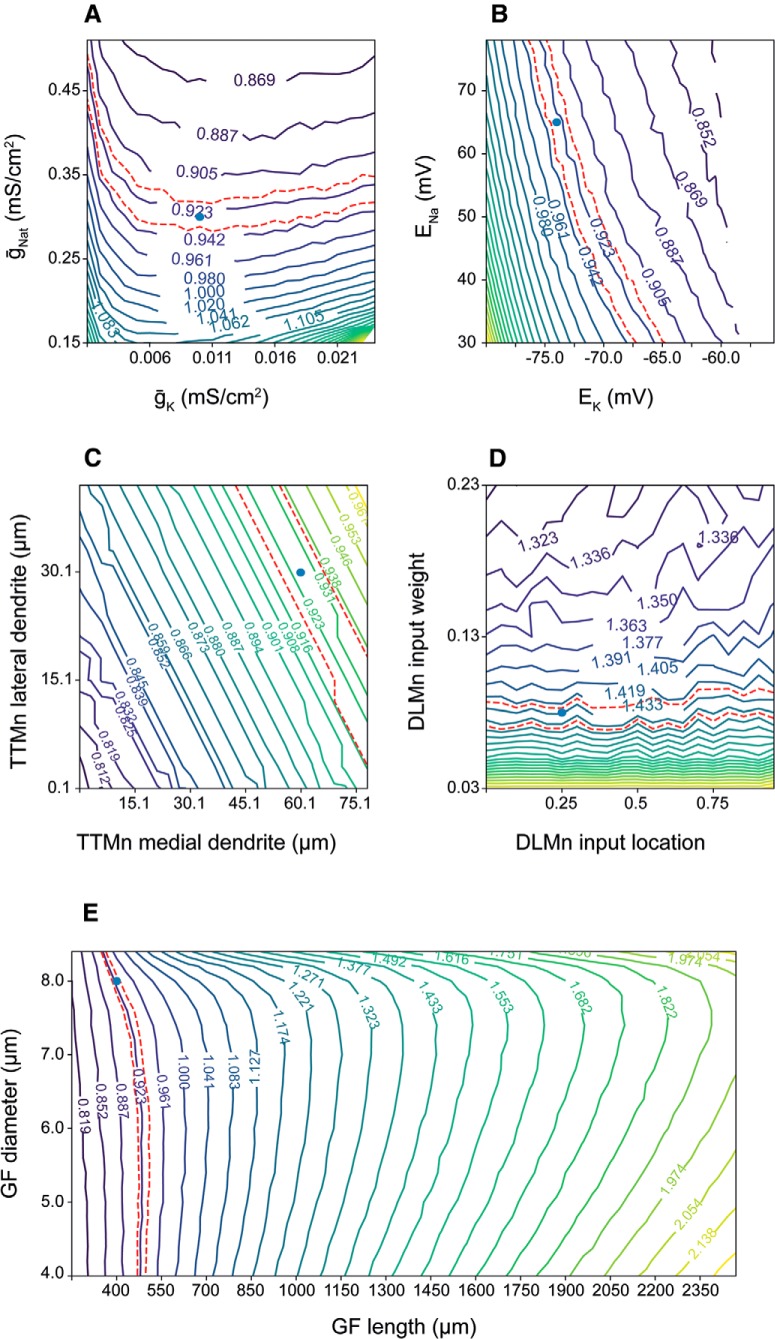
Co-dependency of the response latency on different parameter combinations. ***A***, TTM latency as a function of maximal voltage-gated transient sodium conductance (ḡ_Nat_) and maximal voltage-gated potassium conductance (ḡ_K_). ***B***, TTM latency as a function of sodium reversal potential (E_Na_) and potassium reversal potential (E_K_). ***C***, TTM latency as a function of the TTMn medial dendrite length and TTM lateral dendrite length. ***D***, DLM latency as a function of the PSI-to-DLMn chemical synapse weight, and the synapse location along the DLMn dendrite. ***E***, GF latency as a function of the GF diameter and length. Blue dots represent the values for young and old flies, respectively.

Our model enables predictions of the response latency as a function of arbitrary parameter combinations and may therefore be used as a benchside tool in experimental studies of signal propagation via the GFS. For example, Figure 5*A* shows the TTM latency landscape for voltage-gated sodium and potassium maximal conductances, each affecting the latency in a similar way as shown earlier against g_gap_ (Fig. 3*A*,*B*). Sodium and potassium reversal potentials, functions of intracellular and extracellular ionic concentrations, influence the resting membrane potential, and thus their depolarization reduces the latency to TTM response (Fig. 5*B*). Increase in the TTMn medial and lateral dendrite lengths leads to an increase in membrane load and thus to increased TTM latency (Fig. 5*C*, see also Fig. 4*A–H*), but the medial dendrite length is more influential, since the GFs form synapses with the TTMn on this dendrite (Godenschwege et al., 2002a). The PSI-to-motoneuron contacts are chemical (Tanouye and Wyman, 1980; Allen and Murphey, 2007), so increasing the weight of this synapse naturally shortens the DLM response latency by accelerating the formation of AP in the DLMn as a response to PSI activity; the synapse location on the DLMn dendrite has hardly any influence (Fig. 5*D*). We also tested the impact of the GF length and diameter on response latency through the GFS (Fig. 5*E*). For shorter axons (left side), the optimal diameter (where minimal latency is achieved) is rather small, since the longitudinal AP propagation time is negligible compared to the passive charging of the membrane (see also Fig. 4*A*,*E*). For longer axons (right side), the propagation time becomes more important compared to membrane charging, so the optimal diameter is larger.

In summary, among the tested parameter combinations we identified potassium reversal potential and dendrite length as the parameters with high impact on response latency, with the DLMn input location having a relatively small effect. These results can help guide experiments aimed at manipulating the GFS latency responses.

## Discussion

Our biophysical model of the *Drosophila* GFS accomplished three main things. Firstly, it recapitulated the latency responses previously measured in young and old flies (Augustin et al., 2017). The so called “short-latency responses” are elicited by applying a high-frequency stimulus to the brain, thereby directly activating the GF interneurons and bypassing the presynaptic (sensory) inputs to the GFs (Tanouye and Wyman, 1980; Engel and Wu, 1996). These “stimulus-to-muscle depolarization” response times therefore represent a readout for the functionality of the GFS that includes the GFs, the interneurons and motoneurons downstream of the GFs, as well as the (jump and flight) muscles innervated by the motoneurons (Fig. 1). As the neuromuscular latency is not compromised by age (Augustin et al., 2017), we excluded the muscles (and their respective neuromuscular junctions) from the model, focusing on the period between the brain stimulus and motoneuronal (TTMn and DLMn) AP peak. To be fully explained solely by the properties of gap junctions, our model suggests that the experimentally measured age-related increase in response latency requires a ∼75% reduction in gap junctional conductance (Fig. 2*B*,*C*). While decreased conductance can be caused by either gap junctional loss or dysfunction, this level of attenuation of conductance via gap junctions is within the estimate of the age-associated gap junctional loss in the GFS (Augustin et al., 2017).

Secondly, our model demonstrated the degree to which manipulations of principal membrane ionic conductances can influence the GFS response latencies. Augmentation of the transient voltage-gated sodium conductance in our model (equivalent to an overexpression or hyperactivation of voltage-gated sodium channels *in vivo*) shortened the response latency, with the increase in potassium and leak currents having the expected, opposite, effect (Figs. 3, 5*A*). Considering the critical role of sodium currents in the generation of APs (Elmslie, 2010), the effect of increased Na^+^ conductance is likely due to lowered AP threshold. However, when manipulated within a physiologically relevant range, sodium conductances were unable to revert the latencies from old flies to youthful levels (Fig. 3*A*,*D*).

Thirdly, we showed that anatomic features of the GFS neurons can have a stronger effect on the speed of signal propagation via the circuit (Figs. 4, 5*C*) compared to physiologic parameters. Since the main factor in the model’s response latency is the membrane charging time to AP threshold, any change to the membranal load (e.g., change in membrane surface area as a consequence of changing the length or diameter of a neuronal section) strongly affects the response latency by modulating the cells’ response dynamics to input current. Interestingly, decreasing the diameter of the PSI in old flies resulted in the reversal of response latencies to youthful values. For longer axons such as the GF, however, the propagation velocity may be more important than the membrane charging time (which is influenced by the membranal load; Rall, 2011). Indeed, optimal GF axon diameters scale with the length of the axonal section in determining the GFS response latency such that for short fibers the diameter that produces the minimal latency is smaller than the one for long fibers (Fig. 5*E*). The wide range of values for these anatomic and physiologic parameters shown in the contour maps does not necessarily represent values that are present or measured *in vivo*; it does, however, make it possible to determine, for example, whether the effect of various physiologic and morphologic parameters is continuous or whether there is a threshold beyond which an effect occurs.

Since the bulk of the signal propagation in the circuit is done via long axons and with minimal convergence of inputs, we could use simple, reduced geometry to represent the circuit components. With the addition of ion channel parameters previously characterized in *Drosophila* motoneuron simulations and several literature-based physiologic constraints, our model is likely to faithfully reproduce salient anatomic and physiologic features of the GFS.

The versatile *Drosophila* genetic toolbox allows for time-controlled modulation of relevant membrane conductances in individual GFS neurons and in the whole circuit (Olsen and Wilson, 2008; Jenett et al., 2012). Genetic manipulations of neuronal morphologies, including the size dendritic and axonal sections, are also possible (Scott et al., 2003; Sugimura et al., 2004), although these interventions have not yet been tested in the GFS. These tools can be used to experimentally test the results presented here, with the goal of increasing signal propagation speed through the circuit and furthering our understanding of this escape response system. Although our model is only partially constrained and some of its parameters had to be estimated, it is useful in developing intuition on which circuit elements are likely to have greater influence on the GFS response latency. The model code was made available and can be easily adapted to explore additional parameter combinations in addition to the ones presented here, and updated as more constraints become available.

## Synthesis

Reviewing Editor: Douglas Bayliss, University of Virginia School of Medicine Decisions are customarily a result of the Reviewing Editor and the peer reviewers coming together and discussing their recommendations until a consensus is reached. When revisions are invited, a fact-based synthesis statement explaining their decision and outlining what is needed to prepare a revision will be listed below. The following reviewer(s) agreed to reveal their identity: Mark Beenhakker.

This manuscript was seen by two reviewers, both of whom note that this computational work provides a realistic model of aging-related changes in the Drosphila giant fiber escape circuit, specifically with respect to a documented loss of gap junctions in the circuit; the work generates testable hypotheses and will be of interest to the field. That said, the reviewers individually expressed major concerns with different aspects of the manuscript. In particular, you will note that they recommend: [1] that the paper provide a more complete description of the model architecture (e.g., define segments vs. isopotential compartments); and [2] clarify the parameters that are most salient for understanding aging-related changes (e.g., justify better the emphasis on gap junctions given that changes if EK and dendritic length also affect response latency in the model, but perhaps are not observed to change with age). The full comments from both referees are provided below. Overall, the suggestions deal primarily with improving clarity in the text and figures, and can be addressed without the need for additional experimentation.

Full Reviews

Reviewer #1

Summary

The authors present a purely computational study that aims to systematically identify key parameters that define response latency within the drosophila Giant Fiber System (GFS), a dedicated circuit that elicits a rapid escape response when the insect is threatened. Although both chemical and electrical synapses are found in the GFS, the electrical synapses are thought to significantly contribute to the rapid responses associated with the GFS. Recent behavioral work shows that the response speed slows with age, a phenomenon hypothesized to result from an age-related decline in electrical synapses. Although the study is purely computational, the findings help formulate reasonable, testable hypotheses for future experiments.

The authors should be commended for developing a computational model that appears to generally adhere to known physiological parameters. The authors also present their exploration of the complex parameter space using fairly understandable graphical approaches (e.g. iso-latency plots); although walking the reader through one of the plots might be helpful (see below).

This reviewer's primary concerns are two-fold. The first concerns readability. In the introduction, the authors focus the readers' attention on the importance of electrical synapses in mediating rapid escape responses. The authors also present the hypothesis that slower responses in old flies result from impaired gap junctional communication between the giant fibers and interneurons/motoneurons. Indeed, the authors appear to leverage their computational efforts in testing this hypothesis. However, the authors appear to ultimately conclude that the response latency in the GFS is primarily dictated by the global potassium reversal potential (E-K) and TMMn dendrite diameter. If true, then are the authors arguing that electrical synapse reduction does not drive slower responses in adult animals?

The second concern of the study is that it's not clear to what extent altered E-K and/or TMMn dendrite changes (possibly a 5-fold change!, Fig 5C) are realistic. Have age-related changes in these parameters been measured in flies?

Specific questions and comments are listed below:

1.The response latency from stimulus to action potential in the TTMn and PSI is equivalent in young animals. This result is not surprising because the electrical synapses associated with the GF-TTMn and GF-PSI are modeled with equivalent conductances (135uS). However, when both electrical synapses are similarly reduced to model older animals, then the latency to the PSI response becomes longer than the GF-TTMn response. Why?

2.In the behaving animal, do GF neurons initiate the escape response by producing a single action potential, as appears to be currently modeled in silico? If not and a train of GF spikes is required for escape, then is it possible that increased response latency in old flies is associated with more spike failures, as opposed to an increased duration to reach action potential threshold? Perhaps this possibility represents a subtle difference in conclusions, but do the authors suggest that even GF action potentials in old animals faithfully evoke post-synaptic action potentials, just with longer latencies?

3.From Fig 3, it seems that varying sodium, potassium or leak conductances has little effect on latency when g-gap is low (i.e. the iso-latency lines are fairly flat). I'm having some difficulty understanding this result. It would seem that latencies should become more dependent on these conductances when g-gap is minimized? But the plots appear to show that latencies become more dependent on these ionic conductances when g-gap gets larger. This conclusion seems counterintuitive. Is this reviewer missing something?

4.Regarding Fig 4, to what extent are the ranges in dendrite/axon diameters observed in young and old flies?

5.The authors generally explain their interpretation of the iso-latency plots (e.g. lines 193-202). However, as several iso-latency plots are shown, the reader might benefit if the authors fully explain the significance of the trajectory of the red dotted lines. For example, it might be helpful to point out that similar response latencies can be achieved with high g-Nat/low g-Gap and low g-Nat/high g-Gap. True?

6.The authors conclude that potassium reversal potential and dendrite length are impactful parameters underlying response latency. This conclusion seems at odds with the hypothesis presented in the introduction: reduced gap junction expression underlies prolonged escape response latencies in old animals. Have the authors provided computational evidence that counters their hypothesis? If ionic/morphological changes underlie increased response latencies, then to what extent does E-potassium and/or dendrite morphology change with age?

Minor Comments

1.Perhaps the authors should remind the reader of the definition of gap junction plaque. To the non-expert, it's not initially clear why reduced ShakB plaque size (line 68) is important.

2.The charging times associated with the pre-action potential threshold rising phase of the electrical (GF-PSI and GF-TTMn) synapses appears to be equivalent - perhaps even slower - to the rising phase of the chemical synapse (PSI-DLMn, Fig 2B). Should the rising phase elicited by an electrical synapse be slower than the rising phase elicited by a chemical synapse?

Reviewer #2

Comments to the Authors

In this work the authors describe a simplified model of the giant fiber escape circuit in Drosophila and use it to offer insight into how the documented loss of gap junctions during aging can account for an increase in response latency for escape movements. The effects of structural and membrane conductance parameter's on response latencies are also explored. The work is carefully done and the figures except for Figure 2 illustrate the relevant data very well. The writing in Introduction, Results and Discussion is clear. The findings should be of interested to those analyzing fly escape and other escape circuits and also contributes to our understanding of how gap junctional loss during aging might affect circuit performance.

CONCERNS

1. My major concern is with the description of the model in Methods and in Figure 2. I was very confused by the presentation. My understanding is that the basic unit of realistic neuronal modeling is the isopotential compartment and that this compartment is linked to other compartments via an internal resistance, which can be expressed by Ri in Ohm-cm. The authors identify functional regions such as axon and dendrite as compartments. I find this confusing because you should be defining compartments as the unit of computation (isopotential) and functional regions (axons, dendrites, etc) as sets of similar or near similar compartments. From what I can tell all axonal compartments have the same conductance densities but maybe not the same diameter ("DLMn contains a tapering axon..." and two different DLMn axonal 'compartments' (proximal and distal) are described in Table 1). I am thinking your functional regions consist each of 51 compartments ("All compartments are divided into 51 segments each..."), so you must specify Ri in Ohm-cm. Where is this parameter in Table 1? I must assume that what you are calling segments are isopotential compartments. Please describe the model architecture more carefully so that readers familiar with standard neuronal compartmental modeling can understand it more easily. In Figure 2, please label functional regions (including DLMn distal and proximal axon) and indicate lengths and diameters by drawing to scale. Indicating the true compartments might also be helpful.

2. Lines 17-18: "Understanding how to prevent functional decline of neuronal circuits is of utmost importance from both biological and clinical perspective." This is an overstatement of the significance of the work. Because the model is not designed to offer mechanistic insight to how gap junctions decline with aging, this sentence should be reformulated.

Other:

1. Lines 115-119: I am confused by this description, Please rewrite for clarity.

2. Lines 158-160: I am confused here. Don't you mean TTM and DLM when giving these latencies? Shouldn't you make the logic clearer? TTMn and DLMn latencies increase and so TTM and DLM latencies increase to old fly values?

3. Line 226: Confusing sentence transition here.

4. Lines 331-332: Can you be more specific here?

5. Line 343: "...with the goal of improving the circuit's function..." What is you meaning here? Do you mean how evolution might act? Are you thinking of genetic engineering of flies so they are even more difficult to swat?

6. Please at least consider changing all uses of "electrical synapse" to electrical coupling. It makes the function of gap junctions so much clearer.

7. Some minor typos exist.

## Author Response

Dear Dr Bayliss, We would like to express our gratitude to the reviewers for their patience and insightful input. We have now submitted a modified manuscript taking into consideration the suggestions proposed by the reviewers. Please, see below our point-by-point responses to the reviewers' comments. Best regards.

--------

Reviewer #1

1.The response latency from stimulus to action potential in the TTMn and PSI is equivalent in young animals. This result is not surprising because the electrical synapses associated with the GF-TTMn and GFPSI are modeled with equivalent conductances (135uS). However, when both electrical synapses are similarly reduced to model older animals, then the latency to the PSI response becomes longer than the GF-TTMn response. Why? This effect is due to the difference in morphology between the cells (higher load in the PSI due to long dendrite), which plays a larger role as the inward current is reduced. A clarification of this process is now added to the text (lines 159-161, 165-168).

2.In the behaving animal, do GF neurons initiate the escape response by producing a single action potential, as appears to be currently modelled in silico? If not and a train of GF spikes is required for escape, then is it possible that increased response latency in old flies is associated with more spike failures, as opposed to an increased duration to reach action potential threshold? Perhaps this possibility represents a subtle difference in conclusions, but do the authors suggest that even GF action potentials in old animals faithfully evoke post-synaptic action potentials, just with longer latencies? Indeed, a single action potential in the GF axon initiates flight by producing patterned activity in jump and flight muscles (e.g. Koto et al., Brain Research, 1981). We have now included this reference and explanation in the main text (lines 41-42). The reviewer is also correct to conclude that the postsynaptic (muscle) potentials are normal in old animals, apart from the increased time between the GF stimulus and muscle depolarisation.

3.From Fig 3, it seems that varying sodium, potassium or leak conductances has little effect on latency when g-gap is low (i.e. the iso-latency lines are fairly flat). I'm having some difficulty understanding this result. It would seem that latencies should become more dependent on these conductances when g-gap is minimized? But the plots appear to show that latencies become more dependent on these ionic conductances when g-gap gets larger. This conclusion seems counterintuitive. Is this reviewer missing something? The reviewer's intuition is right here, and it is the nature of the iso-latency maps that is slightly misleading, especially when the lines are very close to each other. A close examination would show that traveling from the blue or orange dots up to the right edge of the maps would result in crossing more or less the same number of iso-latency lines. To make this point clearer we revised Fig 3 and added crosssections through the map for young and old flies, showing the difference in latency as the conductance

parameters change. These show that the effect is more or less similar for young and old flies, with the slope slightly larger for the latter.

4.Regarding Fig 4, to what extent are the ranges in dendrite/axon diameters observed in young and old flies? The measurements of the GF axon diameter (Fig. 5E) range from 4-6 µm (Power 1948), 5 µm (Tanouye and Wyman 1980; Koto 1981) to 8 µm (Augustin 2017). The measured diameters of other GFS neurons (Fig. 4): TTM axons (6 µm, King & Wyman 1980; 8 µm, Blagburn 1999), PSI axons (1.5 µm, Egger 1997; 4 µm, Blagburn 1999, 3-5 µm, King & Wyman 1980). Dendrite lengths for neurons downstream of the GF were estimated from the publications listed in Table 1. Most of the reported diameters were measured in very young flies (up to 10 days old), with no published studies reporting diameter measurements in old flies apart from our own recent study (Augustin et al., 2017, PLoSBiol).

5.The authors generally explain their interpretation of the iso-latency plots (e.g. lines 193-202). However, as several iso-latency plots are shown, the reader might benefit if the authors fully explain the significance of the trajectory of the red dotted lines. For example, it might be helpful to point out that similar response latencies can be achieved with high g-Nat/low g-Gap and low g-Nat/high g-Gap. True? That is correct. We added such a “roadmap” to the text (lines 199-203) to make the iso-latency plots clearer, along with the addition of the 'cross sections' in Fig 3 (see point 3 above).

6.The authors conclude that potassium reversal potential and dendrite length are impactful parameters underlying response latency. This conclusion seems at odds with the hypothesis presented in the introduction: reduced gap junction expression underlies prolonged escape response latencies in old animals. Have the authors provided computational evidence that counters their hypothesis? If ionic/morphological changes underlie increased response latencies, then to what extent does Epotassium and/or dendrite morphology change with age? We have recently reported that reduction in the density of gap junctions is the likely cause of the extended response latency in old flies (Augustin et al., 2017, PLoSBiol); our modelling results (this manuscript) show that the young and old phenotypes can be simulated by changing gap junctional conductance, further supporting this hypothesis. While our basic model of the GFS relies on measured anatomical and physiological circuit parameters, one of the goals of this manuscript was to test the effect of manipulations of other potential effectors of the GFS function. The wide range of parameter values for these effectors shown in the contour maps does not necessarily represent values that are present or measured in vivo; it does, however, make it possible to show the intricate landscape of influences of various physiological and morphological factors on the response latency. These factors, such as dendrite morphology and potassium reversal potential, likely play a minor role in the ageing GFS compared to the age-related loss of gap junctions, but can be modified (genetically or pharmacologicaly) with the goal of preventing the diminishing circuit's function in the GFS of ageing flies and, possibly, other circuits/species. We have added an additional sentence to Discussion (lines 336-340) to clarify this issue.

Minor Comments

1.Perhaps the authors should remind the reader of the definition of gap junction plaque. To the nonexpert, it's not initially clear why reduced ShakB plaque size (line 68) is important. We have now corrected this omission by adding an additional explanation to the sentence (lines 69-70).

2.The charging times associated with the pre-action potential threshold rising phase of the electrical (GFPSI and GF-TTMn) synapses appears to be equivalent - perhaps even slower - to the rising phase of the chemical synapse (PSI-DLMn, Fig 2B). Should the rising phase elicited by an electrical synapse be slower than the rising phase elicited by a chemical synapse? The “current” rise-time of electrical synapses is indeed faster compared to chemical synapses (as the latter involve receptor binding etc.). However, the voltage change presented in Fig 2B strongly depends on the morphology of the cells in question. Since the DLMn is compact compared to the other cells, a comparable current would result in a higher final voltage change (and thus lower latency to threshold). Moreover, the current amplitude in the two cases is in fact quite different.

Reviewer #2

1. My major concern is with the description of the model in Methods and in Figure 2. I was very confused by the presentation. My understanding is that the basic unit of realistic neuronal modeling is the isopotential compartment and that this compartment is linked to other compartments via an internal resistance, which can be expressed by Ri in Ohm-cm. The authors identify functional regions such as axon and dendrite as compartments. I find this confusing because you should be defining compartments as the unit of computation (isopotential) and functional regions (axons, dendrites, etc) as sets of similar or near similar compartments. From what I can tell all axonal compartments have the same conductance densities but maybe not the same diameter (“DLMn contains a tapering axon...” and two different DLMn axonal 'compartments' (proximal and distal) are described in Table 1). I am thinking your functional regions consist each of 51 compartments (“All compartments are divided into 51 segments each...”), so you must specify Ri in Ohm-cm. Where is this parameter in Table 1? I must assume that what you are calling segments are isopotential compartments. Please describe the model architecture more carefully so that readers familiar with standard neuronal compartmental modeling can understand it more easily. In Figure 2, please label functional regions (including DLMn distal and proximal axon) and indicate lengths and diameters by drawing to scale. Indicating the true compartments might also be helpful.

As the reviewer pointed out, the manuscript indeed suffered from a naming inconsistency with regard to the model architecture. Since the term 'compartment' is sometimes used differently in experimental publications (where it is usually referring to a functional subunit) vs modelling publications (where it is usually referring to the basic iso-potential unit of the model), we have decided to avoid it altogether. Instead, we are using the convention used in the NEURON simulator, where the iso-potential units are known as 'segments', and they make up unbranching 'sections' (which also correspond in our model to functional subunits). In the revised manuscript we therefore changed all the references to 'compartments' or 'processes' to 'sections', and improved the description of the architecture in the methods section. The specific axial resistance was indeed omitted from table 1 (as well as the specific membrane capacitance), so these two parameters are now added. In figure 2, functional regions are denoted by dark/light colors (for axons/dendrites respectively), and all section are drawn to scale. We added arrows to denote the distal ends of the axons, from which the recordings in 2B were taken. Explicitly showing all 51 segments in each section would be difficult in such a scale.

2. Lines 17-18: “Understanding how to prevent functional decline of neuronal circuits is of utmost

importance from both biological and clinical perspective.” This is an overstatement of the significance of the work. Because the model is not designed to offer mechanistic insight to how gap junctions decline with aging, this sentence should be reformulated. We have slightly modified the sentence in line with the reviewer's comments. While we do not offer “a mechanistic insight into how gap junctions decline with aging”, we do demonstrate the relevance of other, non-junctional, anatomical and physiological parameters, the manipulation of which can prevent functional decline of the Giant Fiber and, possibly, other neuronal circuits. From the clinical perspective, restoring normal function of a circuit by means of manipulating components that do not lead to its functional decline may be just as effective as restoring the function of the compromised component.

Other:

1. Lines 115-119: I am confused by this description, Please rewrite for clarity. Rewritten to improve clarity (new lines 119-124).

2. Lines 158-160: I am confused here. Don't you mean TTM and DLM when giving these latencies? Shouldn't you make the logic clearer? TTMn and DLMn latencies increase and so TTM and DLM latencies increase to old fly values? Corrected to TTM and DLM as it should have been (new line 164).

3. Line 226: Confusing sentence transition here. Corrected (new lines 239-241).

4. Lines 331-332: Can you be more specific here? We expanded the sentence to make it more specific (now lines 334-335).

5. Line 343: “...with the goal of improving the circuit's function...” What is you meaning here? Do you mean how evolution might act? Are you thinking of genetic engineering of flies so they are even more difficult to swat? We modified the sentence to make it more specific (new line 351-352). In the case of the GFS, the goal would be to prevent the reported increase in response latency and, consequently, improve the escape response triggered by an approaching predator (or a fly swatter!).

6. Please at least consider changing all uses of “electrical synapse” to electrical coupling. It makes the function of gap junctions so much clearer. Since in the case of the GFS the electrical synapses are unidirectional (rectifying), we felt that the term electrical coupling could be misleading as it suggests bidirectional action.

7. Some minor typos exist. We edited the text and removed the typos.
